# Near-surface geophysical and sedimentological analyses for the lower havel inner delta - a dataset of the floodplain lithostratigraphy

**DOI:** 10.1016/j.dib.2025.112372

**Published:** 2025-12-09

**Authors:** Anne Köhler, Marco Pohle, Matteo Bauckholt, Susann Birnstengel, Marie Kaniecki, Birgit Schneider, Ulrike Werban, Christoph Zielhofer

**Affiliations:** a Institute for Geography, Leipzig University, Leipzig, Germany; b Department Monitoring Exploration Technologies, Helmholtz Centre for Environmental Research - UFZ, Leipzig, Germany; c Historical Anthropospheres working group, Leipzig Lab, Leipzig University, Germany

**Keywords:** Sedimentary architecture, Lower havel inner delta, Electromagnetic induction, Electrical resistivity tomography, driving core drilling, Near-surface geophysics

## Abstract

Floodplains are dynamic and sensitive landscapes, shaped by natural hydromorphological processes and centuries of human intervention. Understanding floodplain lithostratigraphy is essential for reconstructing environmental change and supporting floodplain restoration and management. Here, we present a multi-method, high-resolution dataset from the Lower Havel River Inner Delta in Brandenburg (Northeast Germany), combining electromagnetic induction (EMI), electrical resistivity tomography (ERT), and driving core drilling data to characterise near-subsurface sedimentary architecture and sediment composition across multiple spatial scales.

EMI measurements (*n* ≈ 230,000) were conducted across two areas of 120,000 m² and 77,800 m² using a portable three-coil system (CMD-Mini Explorer, GF Instruments) with vertical dipole configuration and coil spacings of 32 cm, 71 cm, and 118 cm. These provided depth-resolved apparent electrical conductivity (ECa) values. Complementary ERT transects (total length: 928 m) were acquired using a multi-electrode DC resistivity system (Resecs, GeoServe) in wenner alpha and dipole-dipole configurations, generating high-resolution resistivity models. EMI and ERT were applied in combination to enable the extrapolation of transect-based geophysical data into spatially continuous models.

To ground-truth the geophysical results, 21 sediment driving core drillings were extracted with a percussion corer (Cobra Pro, Atlas Copco), described in the field (including Munsell colour, grain size, redox features, carbonate and humus content), and analysed in the lab for content of total organic carbon (TOC), total inorganic carbon (TIC), total nitrogen (TN) and total sulfur (TS) and grain size using sieving and X-ray granulometry. The data provide insight into the vertical and lateral extent of peat, clayey and sandy alluvial units, and glaciofluvial basal sediments. All geophysical and lithological data are georeferenced and made available as raw and processed formats.

This comprehensive dataset supports palaeochannel reconstruction, floodplain stratigraphic modelling, and landscape-scale sediment analysis. It is of high relevance to researchers in geomorphology, wetland ecology, soil science, and environmental reconstruction. Moreover, it provides a valuable baseline for long-term monitoring and restoration planning in Central European lowland river systems.

Specifications Table

**Table:** 

Subject	Earth and Environmental Sciences
Specific subject area	Geophysical and sedimentological investigation of floodplain stratigraphy, wetland deposits in a fluvial lowland environment
Type of data	Figures Processed and visualised
Data collection	Driving core drilling was performed using a Cobra Pro (Atlas Copco) and a 60 mm diameter open corer (1 m length). Electromagnetic induction (CMD Mini Explorer) was conducted in vertical dipole mode (VDP) with coil spacings of 32 cm, 71 cm, and 118 cm (exploration depths: 50 cm, 100 cm, 180 cm). Electrical resistivity tomography (ERT) was carried out on four transects: transect1 (259 m, 0.5 m spacing), transect2 (223 m, 1 m), transect3 (207 m, 1 m), and transect4 (239 m, 1 m), all using wenner alpha and dipole-dipole arrays. DGPS measurements used Leica GPS1200 and Topcon HiPER II with a sub-centimetre accuracy.
Data source location	Institution: Leipzig University and Helmholtz Centre for Environmental Research GmbH - UFZ City/Town/Region: Leipzig Country: Germany Sampling location: Lower Havel River Region, Near Lake Gülpe, Brandenburg, Germany Geographical extent (ETRS89, EPSG: 4258): Southernmost point: 52,727,759 N Northernmost point: 52,736,516 N Easternmost point: 12,22,071,889 E Westernmost point: 12,20,990,311 E
Data accessibility	Repository name: Pangaea Title: Geophysical, Sedimentological and Geochemical Data from the Lower Havel Inner Delta (Gülpe Island), Brandenburg (Germany) [dataset publication series] ([1]) Data identification number: https://doi.pangaea.de/10.1594/PANGAEA.983208
Related research article	None

## 1 Value of the Data

• The combined dataset of electromagnetic induction (EMI), electrical resistivity tomography (ERT), and driving core drillings offers high-resolution information on subsurface lithostratigraphy, grain-size distribution, and sediment composition, allowing for a multi-scale characterization of floodplain environments
• These data are valuable for researchers in geosciences, environmental sciences, soil science, and applied fields such as hydrogeology or wetland restoration, due to the standardised field protocols and broad range of sedimentological and geophysical parameters
• The dataset can be reused for comparative studies in similar lowland fluvial settings, particularly in relation to palaeochannel mapping, peat distribution, and stratigraphic modelling
• The georeferenced nature of the dataset enables integration with remote sensing data, GIS-based landscape analyses, and hydrological modelling frameworks
• The dataset provides a reference point for future monitoring campaigns and supports long-term environmental studies addressing changes in sediment dynamics, groundwater-surface water interactions, or floodplain evolution

## 2 Background

**Fig. 1:**
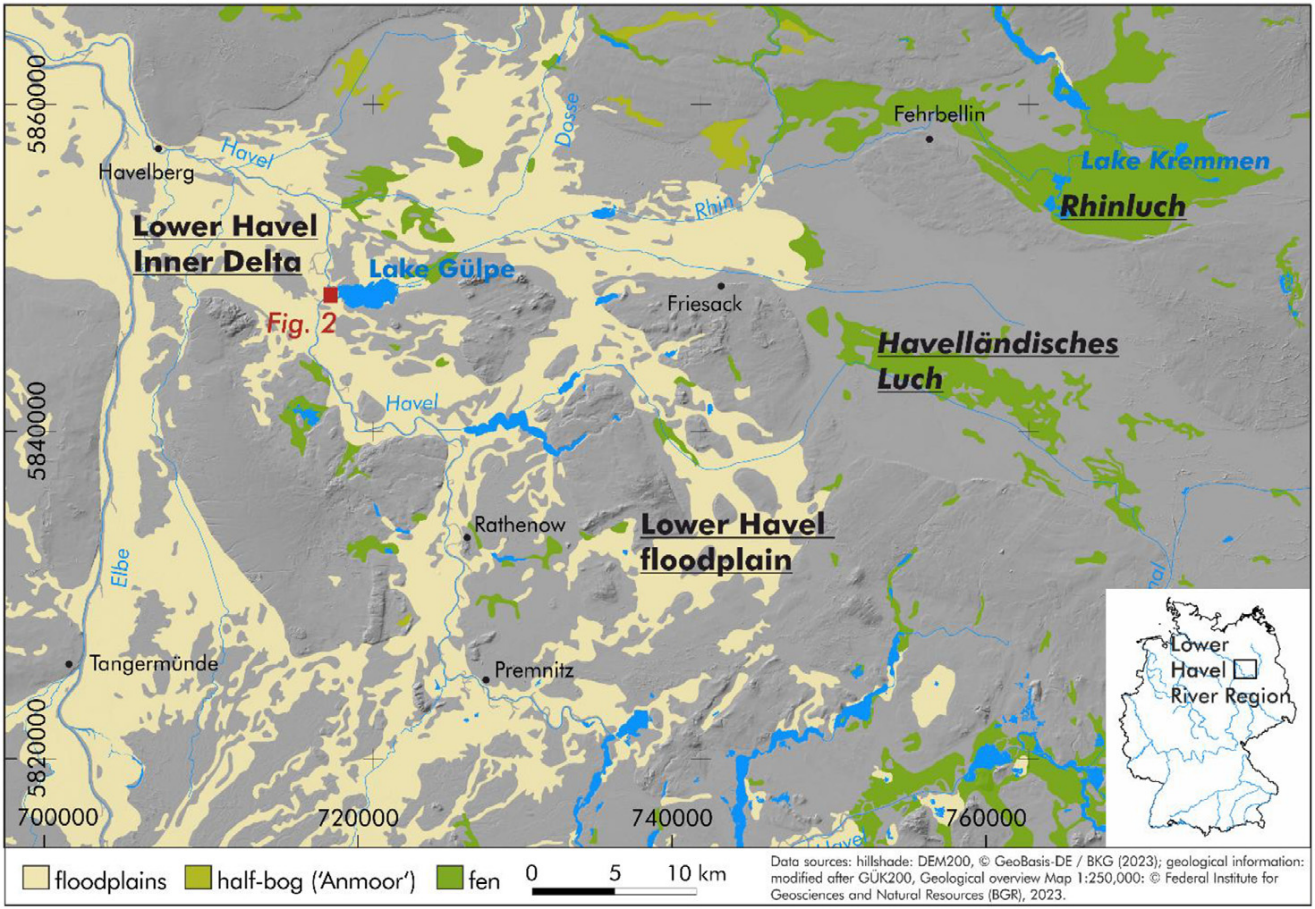
The study area (red rectangle) within the Lower Havel Inner Delta of the Lower Havel River Region.

**Fig. 2:**
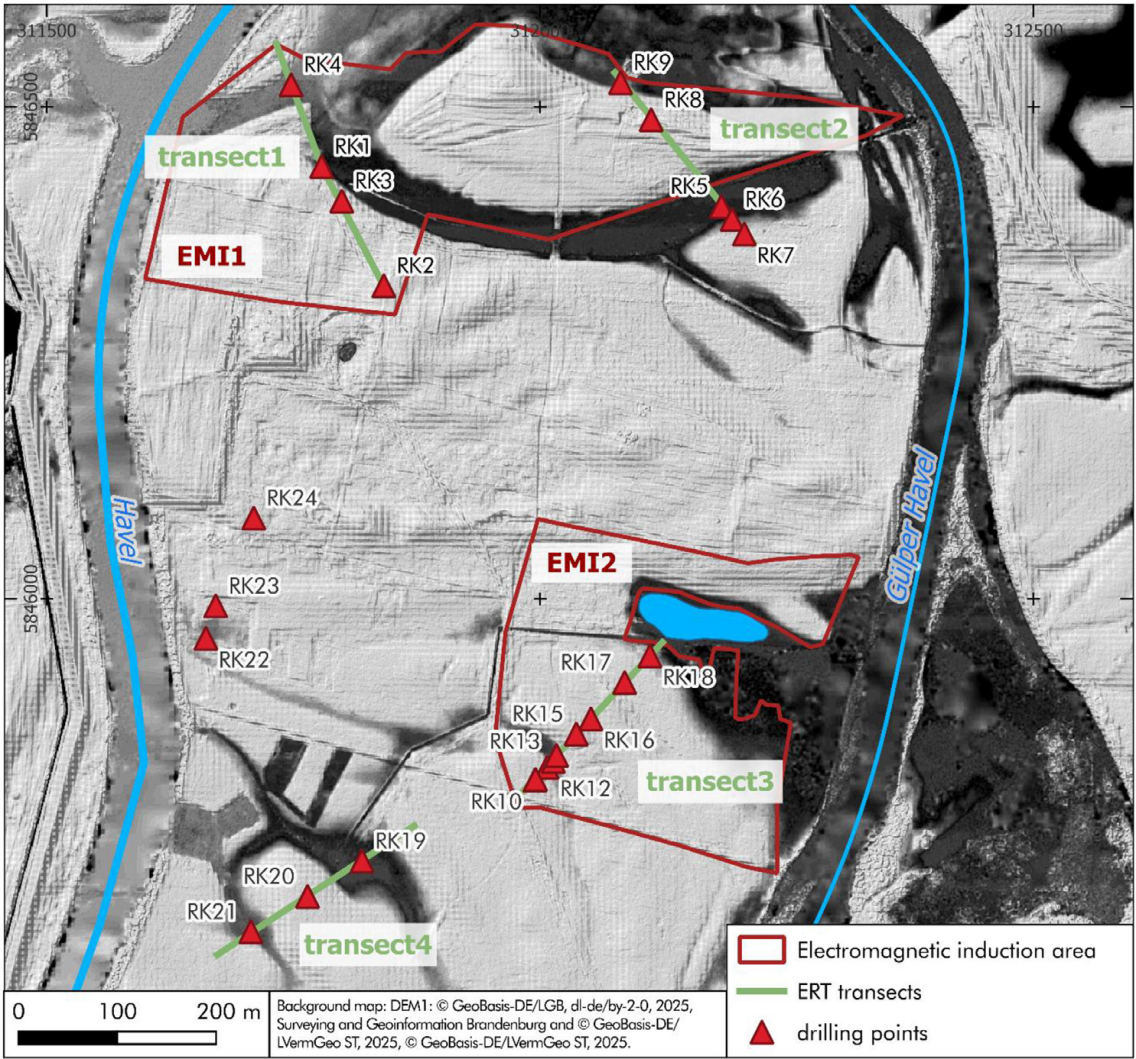
Overview map of the investigated floodplain area in the Lower Havel Inner Delta. Shown are the locations of two EMI survey areas (red outlines), four ERT transects (green lines), and 24 driving core drillings (red triangles). All spatial data are georeferenced and included in the PANGAEA datasets [1-9].

The dataset was compiled to investigate the floodplain lithostratigraphy of the Lower Havel Inner Delta in Northeast Germany (Fig. 1, Fig. 2). The motivation for data collection arose from the need to document spatial patterns of sediment types, grain-size distributions, and stratigraphic sequences in relation to past and present hydromorphological processes.

The data were acquired using a multi-method approach combining high-resolution geophysical techniques (electromagnetic induction and electrical resistivity tomography) with direct driving core drilling. Electromagnetic induction (EMI) and electrical resistivity tomography (ERT) allow for non-invasive characterisation of subsurface electrical properties, which relate to sediment texture and moisture content. The driving core drillings provide ground-truthing and detailed lithostratigraphical description to support the geophysical prospection.

This dataset was generated as part of a broader fluvial geomorphological and environmental reconstruction framework. The data article provides comprehensive access to raw and processed geophysical and sedimentological data, which underpinned the spatial analysis of palaeochannel structures, peat distribution, and floodplain lithostratigraphy.

## 3 Data Description

All raw data referenced in this section are openly available in PANGAEA ([1-9]).

(a) Electromagnetic induction (EMI) data

In the northern part of the investigated Lower Havel Inner Delta, the floodplain subsurface was documented over an area of 120.000 m^2^ using electromagnetic induction (red framed EMI1 area in Fig. 2). In the southern part of the investigated Lower Havel Inner Delta an additional survey of electromagnetic induction covers an area of 77.800 m^2^ (red framed EMI2 area in Fig. 2).

In the EMI1 area, a total of 154,727 data points were interpolated across all three coil spacings (processing details provided in the Experimental Design section). The original dataset includes apparent electrical conductivity (ECa) values ranging from 1.1 to 53.8 mS/m (32 cm coil spacing) ([2]), 0.1 to 54.5 mS/m (71 cm coil spacing) ([3]), and 1.16 to 47.23 mS/m (118 cm coil spacing) ([4]). Table 1 lists the individual parameters as used in the corresponding data tables ([2-7]).

**Table 1:** Overview of EMI measurement parameters as listed in the corresponding data tables ([2-7]).

column	Name of Parameter	Unit	Description
**1**	LATITUDE		
**2**	LONGITUDE		
**3**	UTM Northing, Universal Transverse Mercator	m	
**4**	UTM Easting, Universal Transverse Mercator	m	
**5**	UTM Zone, Universal Transverse Mercator		
**6**	DATE/TIME		
**7**	Location		
**8**	Sample type		type of measurement
**9**	Device type		instrument used for the measurement
**10**	Orientation		coil orientation of the sensor
**11**	Sample comment		additional info on the instrument setup
**12**	Conductivity, electrical	mS/m	

**Fig. 3:**
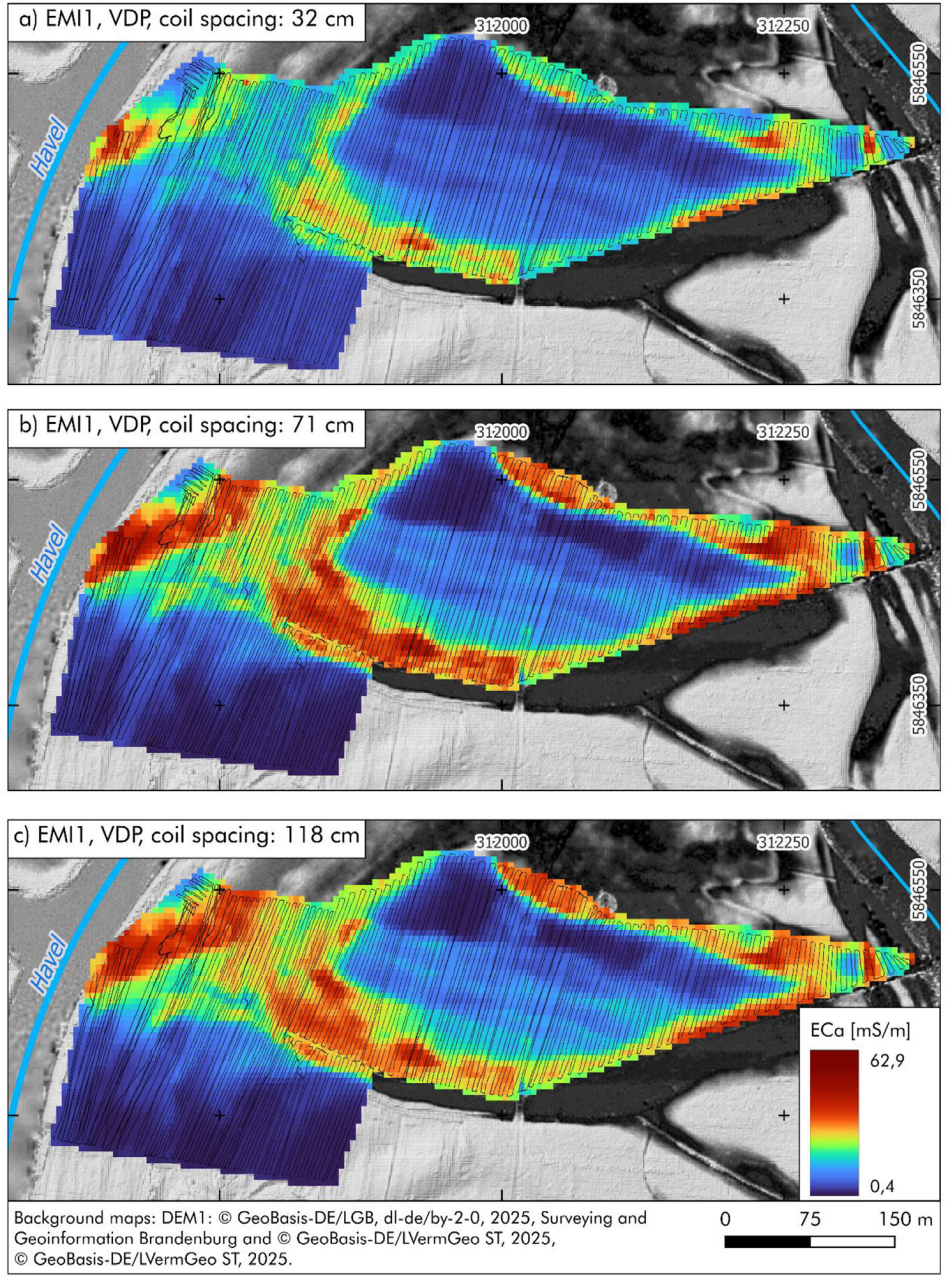
Interpolated apparent electrical conductivity (ECa) values from electromagnetic induction (EMI) measurements in the northern part of the study area (EMI1), recorded in vertical dipole mode (VDP) using three different coil spacings: a) 32 cm (penetration depth: 0.50 m); b) 71 cm (penetration depth: 1.0 m); c) 118 cm (penetration depth: 1.80 m). The small black dots indicate the position of the original EMI measurement points. Raw data are available in PANGAEA [2-4].

Fig. 3a shows the interpolated ECa values for the 32 cm coil spacing, with values ranging from 3 to 46.5 mS/m and low conductivity in the southwestern part of the area. Fig. 3b presents the 71 cm coil spacing, revealing increased conductivity in central areas and a continuation of low values in the southwest. Fig. 3c (118 cm coil spacing) displays ECa values between 1.9 and 44.5 mS/m, following largely the spatial trends observed at Fig. 3b

In the EMI2 area, 76,809 data points were interpolated using the same method as in EMI1. The original ECa values ranged from 0.04 to 66.54 mS/m (32 cm) ([5]), 0.21 to 67.68 mS/m (71 cm) ([6]), and 0.09 to 56.52 mS/m (118 cm) ([7]). Table 1 lists the individual parameters as used in the corresponding data tables ([2-7]).

**Fig. 4:**
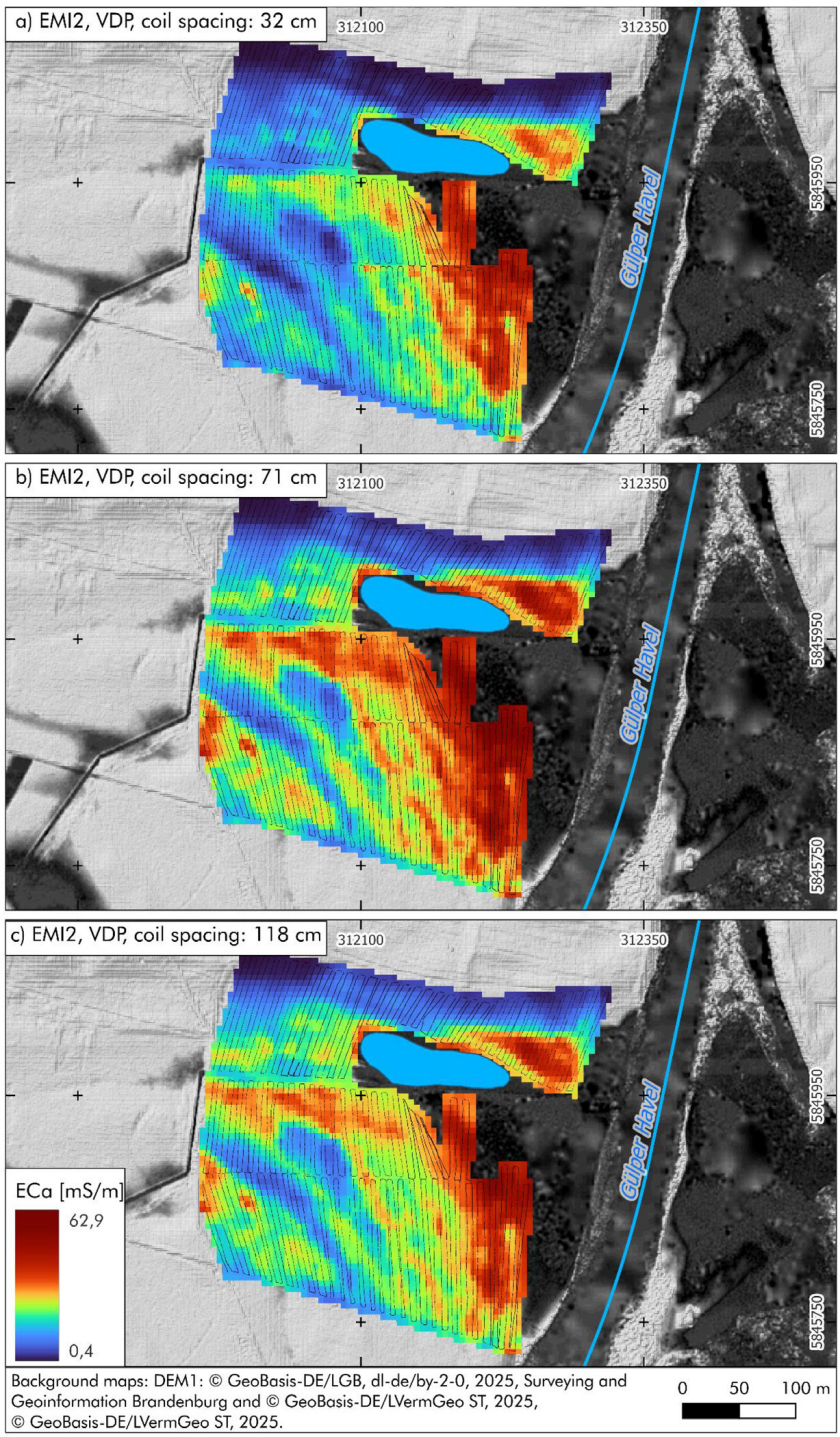
Interpolated apparent electrical conductivity (ECa) values from electromagnetic induction (EMI) measurements in the southern part of the study area (EMI2), recorded in vertical dipole mode (VDP) using three different coil spacings: a) 32 cm (penetration depth: 0.50 m); b) 71 cm (penetration depth: 1.0 m); c) 118 cm (penetration depth: 1.80 m). The small black dots indicate the position of the original EMI measurement points. All raw data are archived in PANGAEA [5-7].

Fig. 4a shows the interpolated values for the 32 cm coil spacing, ranging from 3 to 46.5 mS/m, with low conductivity in the northern and northwestern sections and increasing values from west to east across the central area. Fig. 4b (71 cm coil spacing) indicates higher conductivity across the entire area and enhanced visibility of lateral patterns, with values between 1.9 and 53.6. Fig. 4c (118 cm coil spacing) presents values between 1.9 and 44.5 mS/m, with decreasing contrast, particularly in the eastern high-conductivity zone.

(b) Electrical resistivity tomography (ERT) data

**Fig. 5:**
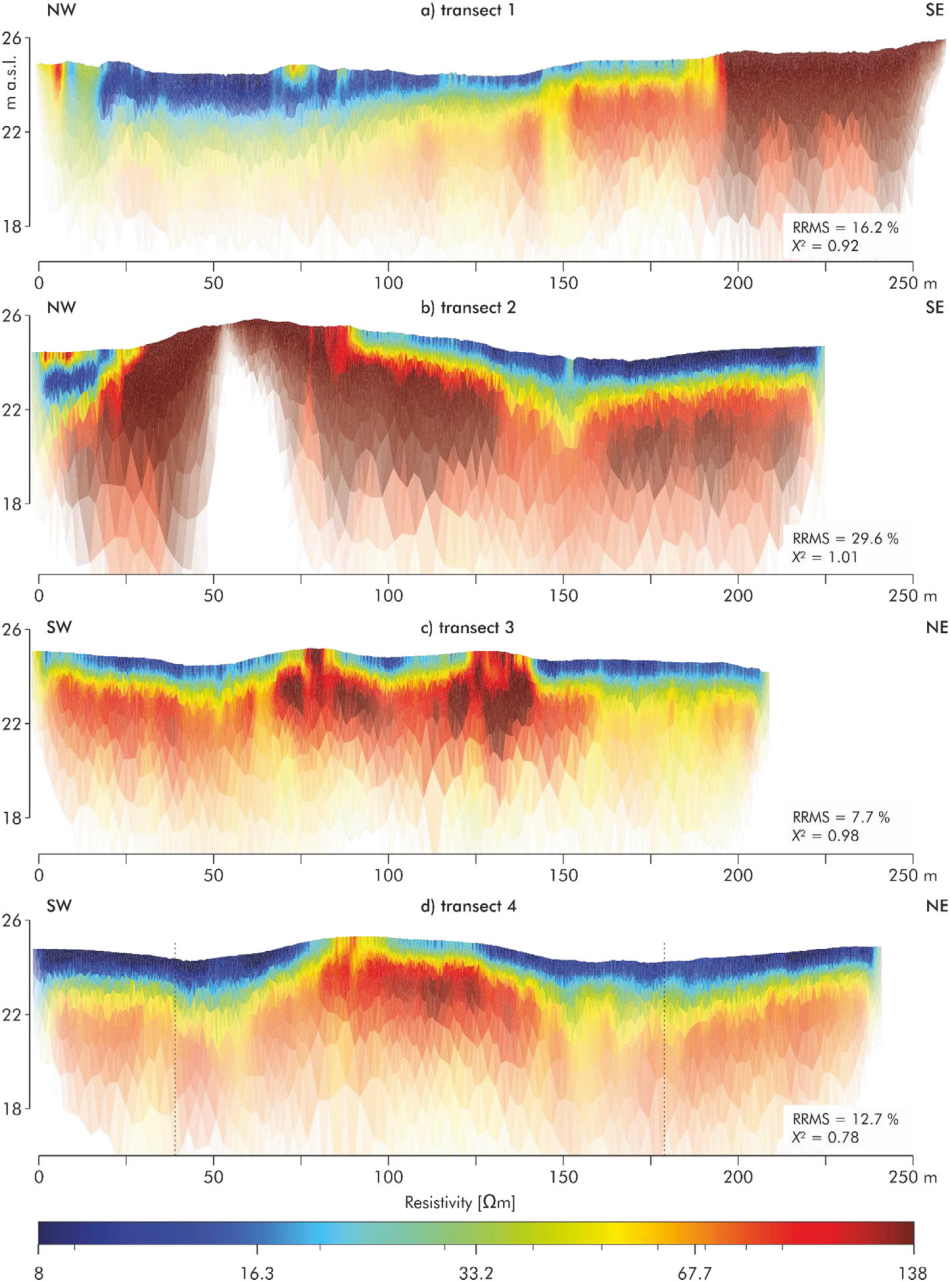
Overview of four ERT transects in the study area. a) transect 1 (NW-SE, 259 m, 0.5 m electrode spacing); b) transect 2 (NW-SE, 223 m, 1 m spacing); c) transect 3 (SW-NE, 207 m, 1 m spacing); d) transect 4 (SW-NE, 239 m, 1 m spacing), the vertical dashed lines indicate the central section highlighted in Fig. 6d The complete ERT datasets are openly accessible via PANGAEA [8].

Within the two areas investigated by EMI (EMI1 and EMI2), targeted transects were established to measure electrical resistivity tomography (ERT) in order to refine the information on subsurface stratigraphy (Fig. 2). Fig. 5 documents the data of the four ERT transects, each illustrating variations in subsurface resistivity in relation to surface topography and floodplain stratigraphy. The ERT transect data are provided in the data file Pohle et al. 2025 ([8]).

ERT transect 1 (Fig. 5a) runs from northwest to southeast and is 259 m long, with an electrode spacing of 0.5 m. Ground elevation ranges between 24.36 and 26.4 m a.s.l (meters above sea level). The northern section exhibits alternating resistivity patterns, overlain by a small zone of high resistivity. Toward the southeast, a distinct layered structure is observed, comprising a surficial low-resistivity unit underlain by more resistive horizons at depth. The thickness of the upper low-resistivity layer gradually decreases along the transect. Toward the end of the transect, the previously layered structure transitions into a more homogeneous and highly resistive unit.

ERT transect 2 (Fig. 5b), also oriented northwest to southeast, is 223 m long with an electrode spacing of 1 m. Ground elevation varies significantly due to topographic undulations, ranging between 24.07 and 25.89 m a.s.l. In the northern section, the resistivity pattern closely resembles that of ERT transect 1, with a near-surface low-resistivity layer. This layer disappears between 25 and 80 m, where it is replaced by a strongly resistive structure. In this part of the transect, the extremely dry soil conditions adversely affected electrode coupling. This resulted in data gaps and subsequent issues during inversion, leading to a relatively high relative root mean square error. From meter 80 onward, low-resistivity material overlies the resistive unit once more. A notable feature at approximately 150 m is a trench-like depression in the otherwise continuous stratification.

ERT transect 3 (Fig. 5c) extends from southwest to northeast over a distance of 207 m, with an electrode spacing of 1 m. The topographic gradient ranges from 24.2 to 25.2 m a.s.l. A high-resistivity unit at depth is overlain by low-resistivity material. Notably, two prominent resistive anomalies interrupt this low-resistivity layer. These anomalies spatially correspond with low-conductivity zones identified in the EMI measurements, suggesting consistency across methods.

**Fig. 6:**
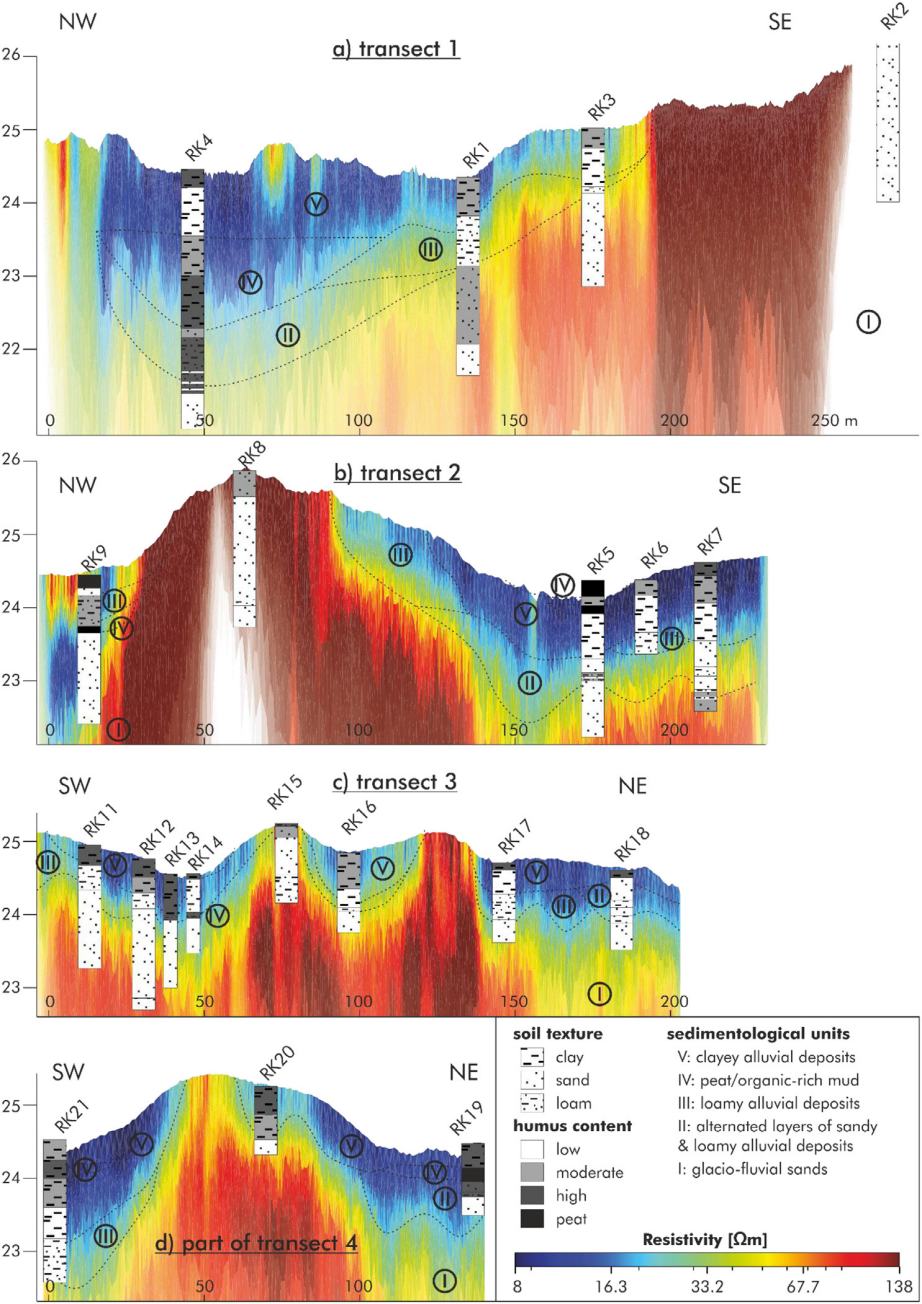
Stratigraphic cross-sections along four transects (1-4) based on 21 driving core drillings (RK1-RK21) integrated with the ERT results from Fig. 5. The sections show ground elevation, sediment texture, humus content, and five sedimentological units: (I) glacio-fluvial sands, (II) alternating sandy and loamy alluvial deposits, (III) loamy alluvial deposits, (IV) peat/organic-rich mud, and (V) clayey alluvial deposits. Dashed lines correspond with the sedimentological units from the driving core drillings. These driving core drilling-based stratigraphy and sedimentological units are documented in PANGAEA [9].

ERT transect 4 (Fig. 5d), also oriented southwest to northeast, is 239 m long with an electrode spacing of 1 m. Ground elevation ranges between 24.15 and 25.3 m a.s.l. The resistivity section shows a near-surface low-resistivity layer (blue to yellow) extending across most of the profile. In the central part of the transect, where surface elevation increases, a zone of higher resistivity (orange to red tones) reaches close to the surface. In the southwestern part around 50 m, as well as between 150 m and 200 m, channel-like patterns are visible: here, the low-resistivity unit extends deeper into the underlying higher-resistivity zone than in the adjacent sections. Two vertical dashed lines at approximately 40 m and 160 m mark the boundaries of the central section shown in Fig. 6, where the data of the driving core drilling are presented.

(c) Stratigraphic cross-sections on the base of driving core drilling data

Fig. 6 presents stratigraphic cross-sections along four transects (1-4) in the study area, derived from 21 driving core drillings (RK1-RK21) in combination with the 4 ERT transects. The profiles show ground surface elevation (in meters above sea level), sediment texture (clay, sand, loam), and humus content (ranging from low to high, including peat). Five sedimentological units are differentiated and color-coded: (I) glacio-fluvial sands, (II) alternating sandy and loamy alluvial deposits, (III) loamy alluvial deposits, (IV) peat/organic-rich mud, and (V) clayey alluvial deposits. Soil texture and humus content were determined based on field observations during coring. The recovered stratigraphical, sedimentological and laboratory data are provided in the data file Köhler et al., in 2025 ([9]). Table 2 shows the different parameters of these data.

**Table 2:** Parameters from sedimentological field recordings and laboratory analyses of the driving core drillings ([9]).

Column	Name of parameter	Unit	Method/Device	comment
1	Event label			
2	Penetration depth	m		
3	Depth, top/min	m		
4	Depth, bottom/max	m		
5	Texture			field-based grain size analysis according to AG Boden, 2024 (Fig. C11, p.255)
6	Grain size classification			field-based grain size class
7	Color code HLS-system		Munsell Soil Color Chart	
8	Color code HLS-system		Munsell Soil Color Chart	Munsell text format
9	Carbonates			estimated carbonat content according to AG Boden 2024 (Tab. C61, p. 272)
10	Humus content			estimated humus content according to AG Boden 2024 (Tab. C32, p. 221)
11	Redoximorphic features			
12	Comment			further information and details about the layers
13	Soil horizon			Classification according to Ad-Hoc-AG Boden 2005 (KA5) and Ad-Hoc-AG Boden 2024 (KA6)
14	Sample ID			
15	Depth, top/min	m		
16	Depth, bottom/max	m		
17	Nitrogen, total	%	Carbon and nitrogen and sulfur (CNS) element analyzer, Elementar, Vario EL Cube	
18	Carbon, total	%	Carbon and nitrogen and sulfur (CNS) element analyzer, Elementar, Vario EL Cube	
19	Carbon, inorganic, total	%	Calcimeter, Royal Eijkelkamp, 08.53	
20	Carbon, organic, total	%		Ct - Canorg
21	Sulfur, total	%	Carbon and nitrogen and sulfur (CNS) element analyzer, Elementar, Vario EL Cube	
22	Size fraction 2.000-0.630 mm, coarse sand	%	Dry sieving	
23	Size fraction 0.630-0.200 mm, medium sand	%	Dry sieving	
24	Size fraction 0.200-0.125 mm, fine sand	%	Dry sieving	
25	Size fraction 0.125-0.064 mm	%	Dry sieving	
26	Size fraction 0.063-0.020 mm, coarse silt	%	Particle Size Analyser, Micrometrics, SEDIGRAPH III 5120	
27	Size fraction 0.020-0.0063 mm, medium silt	%	Particle Size Analyser, Micrometrics, SEDIGRAPH III 5120	
28	Size fraction 0.0063-0.002 mm, fine silt	%	Particle Size Analyser, Micrometrics, SEDIGRAPH III 5120	
29	Size fraction 2-0.63 µm, coarse clay	%	Particle Size Analyser, Micrometrics, SEDIGRAPH III 5120	
30	Size fraction 0.63-0.2 µm, medium clay	%	Particle Size Analyser, Micrometrics, SEDIGRAPH III 5120	
31	Size fraction < 0.2 µm, fine clay	%	Particle Size Analyser, Micrometrics, SEDIGRAPH III 5120	
32	Error, relative	%	Particle Size Analyser, Micrometrics, SEDIGRAPH III 5120	error grainsize

Transect 1 (Fig. 6a), oriented from northwest to southeast, includes four driving core drillings (RK4, RK1, RK3, RK2) and spans elevations between approximately 22.5 and 25.5 m a.s.l. At the base of the profile, Unit I is present in RK4 and RK2, overlaid by Unit II in RK4 and RK1. Unit III appears at RK1 and RK3, followed by a thin layer of Unit IV (peat) at RK4. The uppermost layer throughout much of the transect is composed of clayey alluvial deposits (Unit V), particularly well developed at RK4, RK1, and RK3.

Transect 2 (Fig. 6b), also running NW-SE, contains five driving core drillings (RK9 to RK5) and includes a marked topographic rise at RK8. Unit I is exposed at the base of RK9, while Unit II appears in RK6. Loamy alluvial deposits (Unit III) are present at RK8 and RK9. Peat or organic-rich mud (Unit IV) is observed at RK5 and RK6. The topmost parts of RK5 to RK7, and again RK9, are dominated by Unit V, forming a relatively continuous upper layer.

Transect 3 (Fig. 6c) extends from southwest to northeast and comprises eight driving core drillings (RK11 to RK18). RK10, which displayed identical stratigraphy to RK11, is not shown. The basal layer across most of the transect is formed by glacio-fluvial sands (Unit I), especially prominent in RK15 to RK18. Unit II is found at RK18, overlying the basal sands. Unit III appears at RK17 and RK18. A substantial thickness of peat (Unit IV) is present in the western part of the transect (RK11 to RK14). The surface across all driving core drillings is covered by clayey alluvial deposits (Unit V).

Transect 4 (Fig. 6d), also oriented SW-NE, includes four driving core drillings (RK21, RK20, RK19, RK22). RK19 and RK21 were located in a temporarily inundated area, with the water surface approximately 10-15 cm above ground level at the time of drilling. Unit I forms the basal layer throughout. Unit II is observed beneath RK22, and Unit III is developed at RK20 and RK22. Peat-rich deposits (Unit IV) are documented in RK19 and RK21, consistent with their position in a water-saturated environment. The uppermost unit across all driving core drillings is Unit V.

## 4 Experimental Design, Materials and Methods

The data compiled in these documents were obtained using various methods. This section provides all field data acquisition and processing details. The locations of survey areas and transect alignments are shown in Figs. 2-4 in the *Data Description* section. a) Electromagnetic induction (EMI) for prospecting near-subsurface stratigraphical features

EMI measurements were carried out using a portable coil system (CMD-Mini Explorer, GF Instruments). This instrument operates based on electromagnetic coupling between a transmitter and a receiver coil, where a current induces a primary magnetic field. The penetration depth is determined by the spacing between the coils. In this study, measurements were conducted in vertical dipole mode using coil separations of 32 cm, 71 cm, and 118 cm, resulting in effective penetration depths of approximately 0.5 m, 1.0 m, and 1.8 m, respectively, where 75 % of the cumulative sensitivity is achieved ([10,11]).

The instrument was carried a few centimetres above the ground, and the position of each measurement was continuously recorded using a differential GPS (DGPS), enabling spatial referencing of each ECa value. The locations of individual EMI measurement points are shown in Fig. 3, Fig. 4, which illustrate the spatial coverage of the datasets archived in PANGAEA ([[2], [3], [4], [5], [6], [7]]). The apparent electrical conductivity (ECa) values obtained reflect differences in grain size distribution and/or soil water content. EMI data were collected during two field campaigns in June 2023 and June 2024. All measurements were conducted in the Lower Havel Inner Delta near Lake Gülpe, a fluvially influenced lowland area bordered by the main course of the Havel River and the Gülper Havel.

The ECa data were interpolated using ordinary kriging on a 5 × 5 m grid, corresponding to the approximate 5 m line spacing of the EMI survey transects. Interpolation and visualisation were performed using Golden Software Surfer to generate continuous conductivity maps. The interpolated maps provide insights into the spatial extent of palaeochannels, alluvial clay deposits, and peat accumulations ([12-14]). High conductivity values typically indicate fine-grained sediments and high soil moisture. b) Electric resistivity tomography (ERT) transects for prospecting near-subsurface stratigraphical features

ERT was used as an active geophysical method to investigate the electrical resistivity structure of the subsurface. Recent studies demonstrate the effectiveness of ERT for shallow subsurface characterisation and for linking geoelectrical signatures to lithological heterogeneity ([15,16]). A multi-electrode DC resistivity system (Resecs, GeoServe, Kiel, Germany) was employed to inject a constant current through vertically inserted electrodes arranged along linear transects. Measurements were conducted using wenner alpha and dipole-dipole configurations.

In total, ERT measurements were conducted along four transects with a combined length of 928 m and electrode spacings of 0.5 m or 1.0 m. The individual transect were measured in their entirety as follows: transect1 259 m (0.5 m spacing, 517 electrodes), transect2 223 m (1 m spacing, 221 electrodes), transect3 207 m (1 m spacing, 208 electrodes) and transect4 239 m (1 m spacing, 240 electrodes).

The apparent resistivity was calculated based on the voltage drop between potential electrodes. The data were inverted using **BERT (Boundless Electrical Resistivity Tomography)** ([17]), allowing for depth-resolved resistivity models. The inversion settings were chosen to apply robust data reweighting with a L1 scheme and a regularisation of λ = 40 - 100. This non-destructive method provides high-resolution cross-sectional images of floodplain and wetland deposits ([12,13]). The coordinates and the height of the electrodes were measured with a DGPS (2023: Topcon HiPer II / 2024: Leica GPS1200).

EMI and ERT were applied in combination to integrate high-resolution transect-based subsurface models (ERT) with spatially continuous data (EMI), enabling the extrapolation of point- and line-based information to the larger floodplain area.

(c) Driving core drilling and sedimentological analyses for the validation of the geophysical (EMI, ERT) data - ground truthing

A total of 21 driving core drillings were carried out to investigate the fluvial sediment architecture of the Lower Havel Inner Delta near Lake Gülpe. Coring was conducted using a hand-held percussion drilling system (Cobra Pro, Atlas Copco) equipped with an open corer (60 mm diameter). The driving core drillings were recovered in 1 m segments and described in the field. The exact positions of the drilling points were recorded using a differential GPS (Topcon HiPer II).

Field descriptions included observations of sediment colour (using the Munsell Soil Color Chart), estimated carbonate and humus content, grain size of the fine fraction ([18,19]), redox features, and other stratigraphic characteristics. Stratigraphic differentiation was based on the dominant grain size. Layers were classified as predominantly silty, clayey, sandy, loamy, or as peat (the latter indicating purely organic deposits without siliciclastic components). All driving core drillings were photographed, documented, and sampled at 5-10 cm intervals for subsequent laboratory analyses.

Bulk samples from five selected driving core drillings (RK1, RK3, RK13, RK15, RK17) were freeze-dried, sieved to <2 mm, and weighed. Total carbon (TC), total nitrogen (TN), and total sulfur (TS) contents were measured using a CNS analyzer (Vario EL cube, Elementar). Inorganic carbon (TIC) was determined by calcimeter measurements (Scheibler method, Eijkelkamp). Organic carbon (TOC) was calculated as TOC = TC − TIC.

Grain Size Analysis: Sediment samples were sieved to <2 mm, and 10 g subsamples were treated with 50 ml of 35 % hydrogen peroxide (H₂O₂) and gently heated to remove organic matter. To disperse particles, 10 ml of 0.4 N sodium pyrophosphate solution (Na₄P₂O₇) was added, followed by ultrasonic treatment for 45 min. The sand fraction was analysed by dry sieving and subdivided into four size classes: coarse sand (2000-630 µm), medium sand (630-200 µm), fine sand (200-125 µm), very fine sand (125-63 µm). Finer fractions were determined using X-ray granulometry (XRG) with a SediGraph III 5120 (Micromeritics), including: coarse silt (63-20 µm), medium silt (20-6.3 µm), fine silt (6.3-2.0 µm), coarse clay (2.0-0.6 µm), medium clay (0.6-0.2 µm), fine clay (<0.2 µm).

The full set of raw lithological descriptions and the data of grain size analyses are available at Köhler et al., in 2025 ([9]).

## Limitations

The geophysical measurements in 2023 were conducted under extremely dry conditions, both prior to and during the survey period. These conditions affected the electrode coupling during the ERT measurements, particularly in transect 2 and from approximately 259 m onwards in transect 1.

## Ethics Statement

We confirm that we have read and adhere to the ethical requirements for publication in Data in Brief, and that the present work does not involve human subjects, animal experiments, or any data collected from social media platforms

## CRediT Author Statement

**Anne Köhler:** Conceptualization, Methodology, Validation, Formal Analyses, Investigation, Data Curation, Writing - original Draft, Visualisation, Project Administration, Writing - review and editing; **Marco Pohle:** Conceptualization, Methodology, Software, Validation, Formal Analyses, Investigation, Data Curation, Writing - original Draft, Visualisation, Project Administration, Writing - review and editing; **Matteo Bauckholt:** Methodology, Software, Validation, Formal Analyses, Investigation, Data Curation; **Susann Birnstengel:** Conceptualization, Methodology, Software, Validation, Formal Analyses, Investigation, Data Curation, Writing - original Draft, Visualisation, Project Administration; **Marie Kaniecki:** Formal Analyses, Investigation; **Birgit Schneider:** Methodology, Validation, Formal Analyses, Investigation, Data Curation; **Ulrike Werban:** Conceptualization, Methodology, Software, Validation, Formal Analyses, Investigation, Data Curation, Writing - original Draft, Visualisation, Supervision, Project Administration, Funding acquisition, Writing - review and editing; **Christoph Zielhofer:** Conceptualization, Methodology, Validation, Formal Analyses, Investigation, Data Curation, Writing - original Draft, Visualisation, Supervision, Project Administration, Funding acquisition, Writing - review and editing.

## Data Availability

PANGAEAGeophysical, Sedimentological and Geochemical Data from the Lower Havel Inner Delta (Gülpe Island), Brandenburg (Germany) (Original data).
